# Kinetics of antibody response in critically ill patients with Middle East respiratory syndrome and association with mortality and viral clearance

**DOI:** 10.1038/s41598-021-01083-y

**Published:** 2021-11-19

**Authors:** Yaseen M Arabi, Ali H. Hajeer, Hanan Balkhy, Sameera Al Johani, Musharaf Sadat, Abdulaziz Al-Dawood, Alanoud Abu Taleb, Jesna Jose, Eman Al Qasim, Abdulaziz Al Ajlan

**Affiliations:** 1 Intensive Care Department, King Abdulaziz Medical City, Ministry of National Guard Health Affairs, College of Medicine, King Saud Bin Abdulaziz University for Health Sciences, Riyadh, Kingdom of Saudi Arabia; 2 King Abdullah International Medical Research Center, Riyadh, Kingdom of Saudi Arabia; 3 Department of Pathology and Laboratory Medicine, King Abdulaziz Medical City, Ministry of National Guard Health Affairs, College of Medcine, King Saud Bin Abdulaziz University for Health Sciences, Riyadh, Kingdom of Saudi Arabia; 4 Antimicrobial Resistance, World Health Organization, Geneva, Switzerland; 5 College of Science and Health Professions, King Saud Bin Abdulaziz University for Health Sciences, Riyadh, Saudi Arabia

**Keywords:** Immunology, Diseases, Pathogenesis, Signs and symptoms

## Abstract

The objective of this study is to examine the IgG antibody response in critically ill patients with the Middle East respiratory syndrome (MERS) and to examine the association of early antibody response with mortality and viral clearance. We collected blood samples from 40 consecutive real-time reverse transcription-polymerase chain reaction (rRT-PCR) confirmed critically ill MERS patients on ICU days 1, 3, 7, 14 and 28. MERS-CoV antibodies were detected by enzyme-linked immunosorbent assay (ELISA), using wells coated with MERS-CoV S1 antigen. Patients were admitted to ICU after a median (Q1, Q3) of 9 (4, 13) days from onset of symptoms with an admission Sequential Organ Failure Assessment (SOFA) score of 11 (6.5, 12). Among the study cohort, 38 patients (95%) received invasive ventilation, 35 (88%) vasopressors, 21 (53%) renal replacement therapy and 17 (43%) corticosteroids. Median (Q1,Q3) ELISA optical density (OD) ratio significantly increased with time (p < 0.001) from 0.11 (0.07, 1.43) on day 1; to 0.69 (0.11, 2.08) on day 3, 2.72 (1.84, 3.54) on day 7, 2.51 (0.35, 3.35) on day 14 and 3.77 (3.70, 3.84) on day 28. Early antibody response (day 1–3) was observed in 13/39 patients (33%) and was associated with lower mortality (hazard ratio: 0.31, 95% CI 0.10, 0.96, p = 0.04) but was not associated with faster clearance of MERS-CoV RNA. In conclusion, among critically ill patients with MERS, early antibody response was associated with lower mortality but not with faster clearance of MERS-CoV RNA. These findings have important implications for understanding pathogenesis and potential immunotherapy.

## Introduction

Over 2 decades, there has been 3 emerging coronavirus causing human outbreaks. In 2003, the severe acute respiratory syndrome coronavirus (SARS-CoV-1) spread from China to at least 26 countries in less than a week^1^. In 2012, Middle East respiratory syndrome coronavirus (MERS-CoV) was first identified in Saudi Arabia and continues to appear sporadically causing a severe form of acute respiratory distress syndrome and multiorgan failure that is associated with a mortality of 67%^2,3^. Since December 2019, the Severe acute respiratory syndrome coronavirus 2 (SARS-CoV-2) has caused the largest known pandemic in recent history. Data on the antibody response in MERS patients are limited. Existing data suggest that antibody response MERS-CoV typically is detected in the second and third week after the onset of the infection^4,5^, but little is known about antibody response among critically ill patients and its association with viral shedding and clinical outcomes^6,7^.

The objective of this study is to examine the IgG antibody response in critically ill patients with MERS and to examine the association of early antibody response with mortality and viral clearance. Data on the kinetics of antibody response in critically ill patients with the MERS can be critical for understanding diagnostic testing, seroepidemiology, pathogenesis and possibly passive immunotherapy.

## Methods

### Settings and patients

In this prospective cohort study, we enrolled consecutive critically ill patients with MERS confirmed by real-time reverse transcription-polymerase chain reaction (rRT-PCR) and admitted to the Intensive Care Unit (ICU) at King Abdulaziz Medical City, Riyadh, Saudi Arabia between April 2015 to January 2016.

### Clinical data

Clinical data was collected using standardized case report forms developed by the International Severe Acute Respiratory and Emerging Infection Consortium (ISARIC)^8^. We documented patients' demographic features, underlying comorbidities, duration from the onset of symptom to presentation to the Emergency Room (ER), ICU and intubation. In addition, physiologic parameters and clinical outcomes including mortality (at ICU and hospital discharge, 90 days, 28 days), mechanical ventilation duration, length of stay in the ICU and hospital were also included.

### Laboratory procedures

rRT-PCR and enzyme-linked immunosorbent assay (ELISA) for MERS-CoV were performed at the King Abdulaziz Medical City laboratory. Diagnostic testing for MERS followed the guidelines set by the Saudi Arabian Ministry of Health; nasopharyngeal swabs or sputum samples, if possible, in non-intubated patients and tracheal aspirates or bronchoalveolar lavage in intubated patients were tested by MERS-CoV rRT-PCR which targeted amplification of the upstream E protein (upE gene) and open reading frame 1a^9,10^. In patients with suspected MERS and negative rRT-PCR, testing was repeated at the discretion of the treating teams. For MERS-CoV positive patients, follow-up respiratory samples were collected approximately 1–2 times per week to assess the clearance of viral RNA for infection control purposes. We defined the time to MERS-CoV RNA clearance in respiratory samples in patients as the time from the first performed rRT-PCR after ICU admission until the test was negative on two occasions, without a positive test afterward^3^.

Blood samples were collected prospectively on days 1, 3, 7, 14 and 28 while the patient was in ICU. MERS-CoV antibodies were measured using ELISA (Euroimmun AG, Lubeck, Germany) using wells coated with MERS-­CoV spike protein subunit 1 (S1)^7,11^. Serum samples were diluted (1:100) and incubated with antigens according to the ELISA manufacturer’s instructions. Positive and negative control serum and calibration samples were included. Antibodies were detected by adding peroxidase-labeled rabbit anti-human IgG (Euroimmun AG, Lubeck). Results were reported as the optical density (OD) ratio calculated as the OD value of the patients' sample divided by the calibrator OD value. We used cutoff values recommended by the ELISA kit manufacturer: a ratio of < 0.8 was considered negative, > 0.8 to < 1.1 was considered borderline, and > 1.1 was considered reactive.

### Statistical analysis

Categorical variables were reported as frequencies and percentages, and continuous variables were reported as medians and quartiles 1 and 3 (Q1, Q3). We defined early antibody response as reactive ELISA test on day 1 or 3 of ICU; delayed response was defined as negative ELISA on day 1 and 3 but reactive later (on day 7, 14 or 28); and absent response as negative ELISA on all tested time points. We compared baseline characteristics, physiologic variables and interventions during the ICU stay and clinical outcomes between survivors and non-survivors by day 90 and also between patients with early antibody response to those with delayed or absent response. We compared categorical variables using chi-square test or Fisher's exact test. For continuous variables, the normality distribution was assessed using the Shapiro–Wilk test and based on normality assumption, we compared the variables using the Student t-test or the Mann–Whitney U test whichever was applicable. We compared serial OD ratios between survivors and non-survivors using a mixed linear model. Kaplan–Meier curve for the time to survival and time to MERS-CoV RNA clearance was constructed for patients with early antibody response and patients with delayed or absent response censoring by discharge and at day 90. P values for log-rank tests were reported.

We analyzed the predictors of time to clearance and time to death using Cox proportional hazard model adjusting for age, comorbid conditions, baseline Sequential Organ Failure Assessment (SOFA), and days from onset of symptoms to ICU admission. We also analyzed predictors of early antibody response by multivariate logistic regression adjusting for age, comorbid conditions, baseline SOFA and days from onset of symptoms to ICU admission.

### Ethics approval

The study was approved by Institutional Review Board of the Ministry of National Guard Health Affairs, Riyadh, Saudi Arabia and was carried out in accordance with relevant guidelines and regulations. Informed consent was obtained from patients or their surrogates.

## Results

### Clinical characteristics, ICU course and clinical outcomes

During the study period, a total of 40 critically ill patients with rRT-PCR-confirmed MERS were enrolled with a median age of 58 years (Q1, Q3: 40, 67). Ten patients (25%) were healthcare workers and 25 (63%) had at least one comorbid condition. Patients were admitted to ICU after a median (Q1, Q3) of 9 days (4, 13) from the onset of symptoms with an admission SOFA score median (Q1, Q3) of 11 (6.5, 12). During the ICU stay, 38 (95%) received invasive ventilation, 35 (88%) were on vasopressors, 31 (77.5%) received neuromuscular blockade, 21 (53%) renal replacement therapy and 17 (43%) were given corticosteroids.

Of the enrolled patients, 19 (48%) died by day 90. Non-survivors were older in comparison with survivors [median age of 66 years (Q1, Q3: 57, 76) compared to 51 years (Q1, Q3: 35, 58), p = 0.007], were more likely to have comorbidities [16/19 (84.2%) compared to (9/21(43%), p = 0.007] and had higher SOFA scores [median 11 (Q1, Q3: 10,13) compared to 8 (Q1, Q3: 4,11), p = 0.03]. The median time from onset of symptoms to ICU admission was similar between the two groups. There was no significant difference in the interventions received between the two groups during the ICU stay (Tables 1 and 2).

**Table 1:** Comparison of baseline characteristics and physiologic parameters of patients with Middle East respiratory syndrome (MERS) between non-survivors, survivors and between those with early antibody response (day 1–3) and delayed or absent antibody response.

Variables	All patients N = 40	Non-survivors N = 19	Survivors N = 21	P value	Early antibody response N = 13	Delayed or absent antibody response N = 27	P value
**Demographics**							
Age (yr), median (Q1, Q3)	57.5 (40.0, 66.5)	66.0 (57.0, 76.0)	51.0 (35.0, 58.0)	0.007	55.0 (41.0, 60.0)	58.0 (34.0, 67.0)	0.94*
Body mass index (kg/m^2^), median (Q1, Q3)	28.4 (26.0, 32.8)	27.6 (24.2, 30.5)	30.1 (27.0, 32.9)	0.26	30.0 (26.5, 34.1)	28.3 (25.4, 30.5)	0.46*
Male gender, no. (%)	29 (72.5)	14 (73.7)	15 (71.4)	0.87	9 (69.2)	20 (74.1)	> 0.99
Healthcare worker, no. (%)	10 (25.0)	0 (0)	10 (47.6)	0.002	4 (30.8)	6 (22.2)	0.57
Community acquired, no. (%)	11 (27.5)	6 (31.6)	5 (23.8)		2 (15.4)	9 (33.3)	
Healthcare-associated, no. (%)	19 (47.5)	13 (68.4)	6 (28.6)		7 (53.8)	12 (44.4)	
Days from onset of symptoms to the emergency room, median (Q1, Q3)	4 (2, 5)	3.0 (1.0, 5.0)	4.5 (2.0, 6.0)	0.21	3.0 (2.0, 5.0)	4.0 (2.0, 6.0)	0.54
Days from onset of symptoms to ICU admission, median (Q1,Q3)	9 (4, 13)	11.5 (3.0, 24.0)	7.0 (6.0, 10.0)	0.60	6.0 (6.0, 10.0)	9.5 (4.0, 23.0)	0.28
Days from onset of symptoms to intubation, median (Q1,Q3)	9 (5, 16)	12.5 (3.0, 26.0)	9.0 (5.0, 11.0)	0.67	7.0 (5.0, 9.0)	11.5 (4.5, 24.5)	0.13
**Comorbidities, no. (%)**							
Any comorbidity	25 (62.5)	16 (84.2)	9 (42.9)	0.007	10 (76.9)	15 (55.6)	0.30
Diabetes with chronic complications	5 (12.5)	3 (15.8)	2 (9.5)	0.65	4 (30.8)	1 (3.7)	0.03
Chronic pulmonary disease	8 (20.0)	4 (21.1)	4 (19.0)	> 0.99	4 (30.8)	4 (14.8)	0.40
Renal disease	7 (17.5)	5 (26.3)	2 (9.5)	0.23	1 (7.7)	6 (22.2)	0.39
Chronic cardiac disease	13 (32.5)	6 (31.6)	7 (33.3)	0.91	7 (53.8)	6 (22.2)	0.07
Any malignancy including leukemia or lymphoma or solid tumors	8 (20.0)	8 (42.1)	0 (0.0)	0.001	1 (7.7)	7 (25.9)	0.24
Immunosuppressant use prior to admission	6 (15.0)	4 (21.1)	2 (9.5)	0.40	1 (7.7)	5 (18.5)	0.64
**Physiological parameters on day 1 of admission, median (Q1–Q3)**							
SOFA score	11 (6.5 , 12.0)	11.0 (10.0, 13.0)	8.0 (4.0, 11.0)	0.03	11 (5.0, 11.0)	11 (7.0, 12.0)	0.34*
PaO_2_/FiO_2_ ratio	145.0 (121.4, 191.7)	133.3 (89.3, 168.3)	152.1 (123.6, 200.1)	0.26	166.0 (125.5, 245.3)	138.0 (120.6, 165.0)	0.19
Mean arterial pressure (mmHg)	69.0 (61.0, 75.0)	62.5 (57.0, 70.0)	72.0 (66.0, 80.0)	0.01	69.0 (65.0, 75.0)	69.4 (60.0, 75.0)	0.72
Lactate (mmol/L)	1.4 (0.9, 2.1)	1.8 (0.9, 3.4)	1.3 (1.0, 1.9)	0.66	1.4 (1.1, 1.9)	1.4 (0.9, 2.1)	> 0.99
Creatinine (µmol/L)	73 (60, 210)	112 (60, 172)	70 (63, 252)	0.97	67.0 (60.0, 150.0)	87.5 (66.0, 210.0)	0.44
Bilirubin (µmol/L)	10.6 (8.8, 17.4)	13.9 (9.3, 32.4)	9.4 (7.8, 14.0)	0.27	10.5 (9.0, 17.4)	11.4 (7.5, 21.4)	0.97
Platelet count (× 10^9^/L)	167 (95, 221)	145 (45, 221)	177 (109, 220)	0.32	167.0 (100.0, 220.0)	167.5 (83.0, 253.0)	0.95*
WBC count (× 10^9^/L)	7.2 (4.9, 11.1)	8.5 (4.9, 12.1)	6.7 (5.1, 8.9)	0.57	7.2 (5.4, 9.4)	7.0 (4.9, 11.7)	0.95

SOFA: Sequential Organ Failure Assessment; PaO_2_/FiO_2_ ratio: the ratio of the partial pressure of oxygen to the fraction of inspired oxygen; Q1: first quartile, Q3: third quartile; For all percentages, the denominator is the total number of subjects in the group. For continuous variables, Mann–Whitney U test is used to calculate the p-value except for p values labeled with * indicating the use of Students t test. For categorical variables, Fisher's exact test is used to calculate the P-value.

**Table 2:** Main interventions and outcomes of patients with Middle East respiratory syndrome (MERS) among non-survivors, survivors and those with early antibody response and delayed or absent antibody response.

Variables	All patients N = 40	Non-survivors N = 19	Survivors N = 21	P-value	Early antibody Response N = 13	Delayed or absent antibody response N = 27	P value
Mechanical ventilation, no. (%)	38 (95.0)	19 (100.0)	19 (90.5)	0.49	13 (100.0)	25 (92.6)	> 0.99
Neuromuscular blockade, no. (%)	31 (77.5)	16 (84.2)	15 (71.4)	0.46	9 (69.2)	22 (81.5)	0.44
Nitric oxide, no. (%)	5 (12.5)	3 (15.8)	2 (9.5)	0.49	3 (23.1)	2 (7.4)	0.42
Prone positioning, no. (%)	9 (22.5)	4 (21.1)	5 (23.8)	0.85	4 (30.8)	5 (18.5)	0.63
Vasopressors, no. (%)	35 (87.5)	18 (94.7)	17 (81.0)	0.35	13 (100.0)	22 (81.5)	0.15
Antivirals, no. (%)	32 (80.0)	15 (78.9)	17 (81.0)	> 0.99	12 (92.3)	20 (74.1)	0.24
Corticosteroids, no. (%)	17 (42.5)	9 (47.4)	8 (38.1)	0.55**	7 (53.8)	10 (37.0)	0.31**
Renal replacement therapy, no. (%)	21 (52.5)	9 (47.4)	12 (57.1)	0.54**	7 (53.8)	14 (51.9)	0.91**
ICU mortality, no. (%)	19 (47.5)	19 (100.0)	0 (0.0)	< 0.0001**	4 (30.8)	15 (55.6)	0.14**
Hospital mortality, no. (%)	19 (47.5)	19 (100.0)	0 (0.0)	< 0.0001**	4 (30.8)	15 (55.6)	0.14**
28-day mortality, no. (%)	12 (30.0)	12 (63.2)	0 (0.0)	< 0.0001**	3 (23.1)	9 (33.3)	0.72
90-day mortality, no. (%)	19 (47.5)	19 (100.0)	0 (0.0)	< 0.0001**	4 (30.8)	15 (55.6)	0.14**
ICU length of stay (days), median (Q1, Q3)	18.0 (9.0, 24.0)	12.0 (8.0, 22.0)	20.5 (17.0, 36.0)	0.01	24.0 (18.0, 43.0)	12.5 (8.0, 21.0)	0.005
Hospital length of stay (days), median (Q1, Q3)	32.0 (23.5, 47.0)	27.0 (20.0, 32.0)	43.0 (29.0, 63.0)	0.002	41.0 (29.0, 62.0)	28.0 (21.0, 45.0)	0.17
Mechanical ventilation duration (days), median (Q1, Q3)	15.0 (10.5, 23.0)	12.5 (9.0, 21.0)	17.0 (11.0, 35.0)	0.19	21.0 (14.0, 35.0)	12.0 (7.0, 20.0)	0.01
**Length of stay among survivors**							
ICU length of stay among survivors (days), median (Q1, Q3)	20.5 (17.0, 36.0)	–	20.5 (17.0, 36.0)	–	29.0 (23.0, 57.0)	17.0 (8.0, 22.0)	0.03*
Hospital length of stay among survivors (days), median (Q1, Q3)	43.0 (29.0, 63.0)	–	43.0 (29.0, 63.0)	–	43.0 (35.0, 67.0)	42.0 (25.5, 62.5)	0.32
Mechanical ventilation duration among survivors, median (days), (Q1, Q3)	17.0 (11.0, 35.0)	–	17.0 (11.0, 35.0)	–	26.0 (14.0, 43.0)	15.0 (7.0, 19.0)	0.11

ICU: Intensive care unit; Q1: first quartile, Q3: third quartile. For all percentages, the denominator is the total number of subjects in the group.

For continuous variables, Mann–Whitney U test is used to calculate the p-value except for p values labeled with * indicating the use of Students t test.

For categorical variables, Fisher's exact test is used to calculate thep-value except for *p* values labeled with **indicating the use of Chi-square test.

### Antibody response

**Figure 1:**
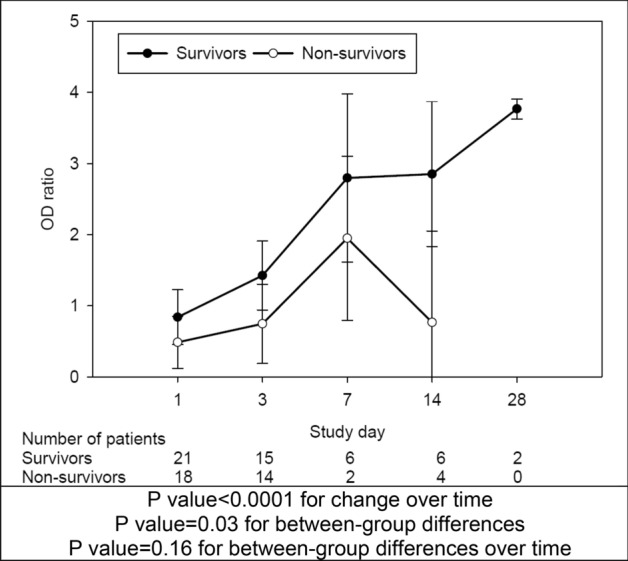
The antibody (IgG) response to Middle East respiratory syndrome (MERS) coronavirus among critically ill patients. Results are expressed as optical density ratio (OD ratio). To account for the fact that repeat antibody testing was not performed after ICU discharge, we carried forward the OD ratio up to day 28 or death whichever came first. Box plots are displayed for the ranks with medians and quartiles 1 and 3. The error bars refer to 1.5*IQR.

Early antibody response (day 1–3) was observed in 13/39 patients (33%). Median (Q1, Q3) ELISA OD ratio significantly increased with time (p < 0.001) from 0.11 (0.07, 1.43) on day 1; to 0.69 (0.11, 2.08) on day 3, 2.72 (1.84, 3.54) on day 7, 2.51 (0.35, 3.35) on day 14 and 3.77 (3.70, 3.84) on day 28. OD ratio over the 28 days of observation was significantly higher among survivors compared to non­ survivors (p = 0.03) (Fig. 1).

### Predictors of antibody response

Stepwise multivariate logistic regression showed that none of the variables entered in the model were statistically significant predictors of early antibody response.

### Association of antibody response and clinical outcomes

**Figure 2:**
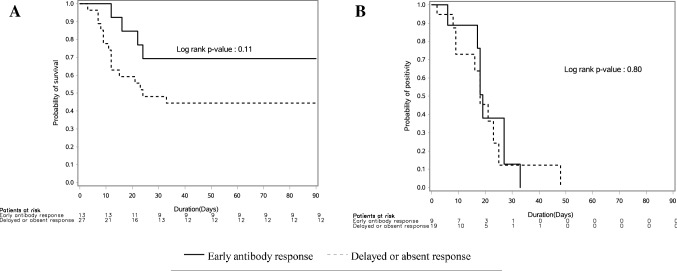
(**A**) Time to survival for critically ill patients who had an early antibody response (Day1-3) and those with delayed or absent response (censored at hospital discharge and at day 90, whichever comes first). (**B**) Time to clearance of the Middle East respiratory syndrome coronavirus (MERS-CoV) RNA from respiratory specimens as tested by the real-time reverse transcription polymerase chain reaction (rRT-PCR) among patients with early antibody response (Day1-3) and those with delayed or absent response.

There was no difference in the time to survival (log rank p value = 0.11) between patients with early antibody response and those with delayed or absent response (Fig. 2A).

Stepwise Cox regression analysis showed that early antibody response was independently associated with lower 90-day mortality [hazard ratio (HR): 0.31, 95% CI 0.10, 0.96, p = 0.04] while any comorbidity associated independently with increased mortality (HR 5.00, 95% CI 1.42, 17.62, p = 0.01) (Table 3).

**Table 3:** Stepwise multivariate models to examine the independent predictors of 90-day mortality, early antibody response* and viral clearance among critically ill patients with Middle East Respiratory Syndrome (MERS).

Variables	Odds ratio (CI)	P-value
**Early antibody response**		
Any comorbidity	4.19 (0.85, 20.71)	0.08
Days from onset of symptoms to ICU admission (for each day increase)	0.91 (0.83, 1.00)	0.06

	Hazard ratio (CI)	
**90-day mortality**		
Early antibody response	0.31 (0.10, 0.96)	0.04
Any comorbidity	5.00 (1.42, 17.62)	0.01
**MERS-CoV RNA clearance**		
Any comorbidity	0.12 (0.04, 0.39)	0.0004

*Early antibody response = positive on day 1 or day 3; CI: Confidence Interval; ICU: Intensive care unit.

All analyses were adjusted for age, any comorbidity, Sequential Organ Failure Assessment (SOFA) and days from onset of symptoms to ICU admission.

For MERS-CoV RNA**,** hazard ratio < 1 signifies delay in clearance.

### Association of antibody response and MERS-CoV RNA clearance

There was no difference in the time to clearance (log-rank p value = 0.80) between patients with early antibody response and  those with delayed or absent response (Fig. 2B). Stepwise Cox regression analysis showed that the early antibody response was not associated with faster MERS-CoV RNA clearance, and that presence of comorbidities was independently associated with delayed viral clearance (HR 0.12, 95% CI 0.04, 0.39, p = 0.0004) (Table 3).

## Discussion

Our study demonstrated that the majority of patients with MERS mounted an IgG antibody response in the first 4 weeks of critical illness. Early antibody response was associated with lower mortality but not with faster clearance of MERS-CoV RNA from respiratory specimens.

Although one third of patients became seropositive within 3 days of ICU admission, it took 28 days for all patients to become seropositive. This finding suggests that serology may not help in diagnosing acute MERS but may help in identifying with a recent history of the disease. Other studies demonstrated similarly that antibody responses to MERS-CoV were detected 2–3 weeks after symptoms^4,5^. Similar results were seen in COVID-19, it took patients about a month from the start of symptoms to become all seropositive^12^.

Our study demonstrates that survivors had higher specific IgG responses than non-survivors and that early antibody response was independently associated with lower mortality among critically ill patients with MERS. This has implications for possible antibody-based immune therapies in early MERS. Of note, a study demonstrated that the development of serum antibody responses in non-survivors during the second and third week of MERS was not sufficient for patient recovery or virus clearance^13^.

We did not observe an association between early antibody response and faster MERS-CoV RNA clearance. A study on 37 patients with MERS showed that the levels of IgG and neutralizing antibodies were weakly and inversely correlated with lower respiratory tract viral loads and that the presence of antibodies did not lead to the elimination of virus from the lower respiratory tract^4^. The dissociation between clinical response and MERS-CoV clearance was observed recently also in the MIRACLE (MERS-CoV Infection treated with a combination of lopinavir–ritonavir and interferon-β1b) trial. In this trial, treatment with lopinavir–ritonavir and interferon-β1b significantly reduced mortality but it did not result in a faster MERS-CoV RNA clearance^14^. Our virologic findings are limited by the lack of data on quantitative viral RNA detection or viral cultures from lower respiratory tract specimens. In a study on a SARS-CoV-2 non-human primate model, early antiviral therapy with remdesivir therapy was greater on infectious virus recovery in bronchoalveolar lavages (BALs) than on viral RNA detection in BALs or upper respiratory tract samples^15^. These data suggest that prolonged MERS-CoV RNA detectability is an insensitive surrogate for clinical disease progression.

Our study has several strengths and limitations. Strengths include being a prospective study in which serial sampling of patients with MERS was carried out in a standardized manner. The number of critically ill patients enrolled is relatively large for a rare disease like MERS. Limitations include the lack of use of neutralization assay, and lack of long term follow up.

## Conclusions

The majority of critically ill patients with MERS demonstrate IgG antibody response in the first 4 weeks of illness. Early antibody response was associated with lower mortality but not with faster clearance of MERS-CoV RNA from respiratory specimens. These findings have important implications for understanding pathogenesis and potential immunotherapy.
